# White Matter Abnormalities and Cognitive Deficit After Mild Traumatic Brain Injury: Comparing DTI, DKI, and NODDI

**DOI:** 10.3389/fneur.2022.803066

**Published:** 2022-03-10

**Authors:** Sihong Huang, Chuxin Huang, Mengjun Li, Huiting Zhang, Jun Liu

**Affiliations:** ^1^Department of Radiology, The Second Xiangya Hospital, Central South University, Changsha, China; ^2^MR Scientific Marketing, Siemens Healthcare Ltd., Wuhan, China; ^3^Department of Radiology Quality Control Center, Changsha, China; ^4^Clinical Research Center for Medical Imaging in Hunan Province, Changsha, China

**Keywords:** mild traumatic brain injury, cognitive function, loss of concussion, DTI, DKI, NODDI

## Abstract

White matter (WM) disruption is an important determinant of cognitive impairment after mild traumatic brain injury (mTBI), but traditional diffusion tensor imaging (DTI) shows some limitations in assessing WM damage. Diffusion kurtosis imaging (DKI) and neurite orientation dispersion and density imaging (NODDI) show advantages over DTI in this respect. Therefore, we used these three diffusion models to investigate complex WM changes in the acute stage after mTBI. From 32 mTBI patients and 31 age-, sex-, and education-matched healthy controls, we calculated eight diffusion metrics based on DTI (fractional anisotropy, axial diffusivity, radial diffusivity, and mean diffusivity), DKI (mean kurtosis), and NODDI (orientation dispersion index, volume fraction of intracellular water (Vic), and volume fraction of the isotropic diffusion compartment). We used tract-based spatial statistics to identify group differences at the voxel level, and we then assessed the correlation between diffusion metrics and cognitive function. We also performed subgroup comparisons based on loss of consciousness. Patients showed WM abnormalities and cognitive deficit. And these two changes showed positive correlation. The correlation between Vic of the splenium of the corpus callosum and Digit Symbol Substitution Test scores showed the smallest *p*-value (*p* = 0.000, *r* = 0.481). We concluded that WM changes, especially in the splenium of the corpus callosum, correlate to cognitive deficit in this study. Furthermore, the high voxel count of NODDI results and the consistency of mean kurtosis and the volume fraction of intracellular water in previous studies and our study showed the functional complementarity of DKI and NODDI to DTI.

## Introduction

The considerable rate of mild traumatic brain injury (mTBI) (1) and the high incidence of post-concussion symptoms such as cognitive deficits and behavioral and emotional changes (2, 3) impose a burden on society. Diffuse axonal injury is thought to be the predominant pathological mechanism underlying mTBI (4–7), and increasing evidence has shown that abnormalities in white matter (WM) caused by mTBI can affect post-concussion symptoms (8, 9). As a result, visualization of WM integrity has become a key part of clinical research.

The Gaussian diffusion of water that diffusion tensor imaging (DTI) assumes within a single microstructural compartment is insensitive to the complexity of the WM microstructure (10). Perhaps as a result, there has been controversy over both the direction and the magnitude of diffusion abnormalities in prior studies on WM tracts (11). Although the injury severity, injury type, time since injury, and sample type may cause inconsistencies between studies, the lack of consensus suggests fundamental limitations of DTI for detecting specific damage in mTBI.

Diffusion kurtosis imaging (DKI), (12) an advanced diffusion MRI technique based on the non-Gaussian diffusion of water, is considered to reflect diffusion in biological tissues more effectively than DTI, especially in brain areas with high tissue heterogeneity. Therefore, DKI might be sensitive to complex tissue changes following mTBI that might not be captured by DTI (13, 14). The most common DKI parameter is mean kurtosis (MK), the average of the diffusion kurtosis along all diffusion directions. However, DTI and DKI are both based on the “signal representations” approach, which lacks specificity and remains an indirect characterization of microstructure.

Neurite orientation dispersion and density imaging (NODDI) is a more advanced multi-compartment diffusion model (15, 16); this technique, based on a “tissue model,” allows the estimation of biologically relevant parameters, which has been validated in the histology of animal and human brain and also used in other disease (17, 18). NODDI leverages recent progress in high-performance magnetic field gradients for MRI scanners that can achieve diffusion-weighting factors much higher than the standard *b* = 800–1,200 s/mm^2^ for DTI and can therefore probe complex non-Gaussian properties of WM diffusion. NODDI uses seven parameters to measure the properties of three microstructural environments: intracellular, extracellular, and free water. It constrains four diffusivity parameters to stabilize the fitting procedure, such that the following three parameters remain: (1) the orientation dispersion index (ODI) of neurites, which is higher in loosely organized WM and lower in tracts with largely parallel fiber bundles; (2) the volume fraction of intracellular water (Vic), which represents the relaxation-weighted volume fraction of the intracellular compartment within WM and ranges from 0 to 1; and, finally, (3) the volume fraction of the isotropic diffusion compartment (Viso), which represents the free water content within the tissue (15, 16).

To date, a few studies have investigated DKI and NODDI in patients with mTBI (19–25). Some of these studies are based on specific samples (athletes or military personnel) who are prone to repeated injury (21, 22, 24). However, directly recruiting consecutive patients from the emergency room may be the best strategy to obtain a representative sample that best generalizes to the overall population of mTBI patients (26).

Loss of consciousness (LOC) is one of the diagnostic criteria for mTBI patients; LOC and the duration of unconsciousness are issues of clinical concern in the acute management of mTBI patients. Patients with LOC have increased inflammation and pain (27). Furthermore, animal models (28, 29) and clinical studies (30–32) have revealed a correlation between LOC and WM integrity. To date, however, there has been no research on the relation between LOC and WM abnormalities detected by DKI or NODDI.

Therefore, the three primary goals of this study are as follows: (1) to investigate whether there is any WM abnormality after mTBI in the acute stage by using DTI, DKI, and NODDI; (2) to determine whether and to what extent LOC affects changes in WM and cognitive function after injury; and (3) to explore the correlation between WM changes and cognitive function after mTBI.

## Materials and Methods

### Participants

This study included 32 mTBI patients and 31 healthy controls (HCs). mTBI patients were enrolled from the emergency department of the Second Xiangya Hospital, Central South University from June 2019 to December 2019. Approval was granted by the Ethics Committee of the Second Xiangya Hospital, Central South University. All participants provided written informed consent. The inclusion criteria for mTBI patients were based on the World Health Organization's Collaborating Center for Neurotrauma Task Force: (1) a Glasgow Coma Scale score of 13–15; (2) any of the following: (a) confusion or disorientation; (b) LOC ≤ 30 min; (c) posttraumatic amnesia <24 h; (d) transient neurological abnormalities (focal signs or seizure); or (e) intracranial lesion not requiring surgery; and (3) a time frame within 7 days after the onset of mTBI. The exclusion criteria were as follows: (1) age below 18 or above 60 years; (2) presence of severe psychiatric disease, severe somatic disease, or drug abuse; (3) history of complicated mild, moderate, or severe TBI or other diseases associated with brain pathology; (4) structural abnormality on neuroimaging (CT and MRI); and (5) contraindications for MRI. HCs were recruited through WeChat Moments and fulfilled the same exclusion criteria as mTBI patients. HCs were recruited from August 2019 to September 2020.

All subjects were clinically evaluated in detail, and psychiatric evaluations were performed in a face-to-face interview by trained staff. The clinical characteristics included the following: age, sex, education, injury time, cause of injury, postinjury symptoms, injury, and MRI scan interval. In the mTBI group, subjects were divided into two subgroups based on whether LOC occurred. Nine patients had a witnessed period of LOC, and then we matched the patients without LOC one by one as the non-LOC group to do the subgroup comparison. The demographic characteristics are presented in Tables 1, 2.

**Table 1:** Demographic and clinical characteristics of mTBI patients and HCs.

	**mTBI**	**HCs**	***t*/Z/c2**	***p***
*N*	32	31		
Sex	M:17; F:15	M:14; F:17	0.4	0.62
Age (y)	32.41 ± 13.29	37.77 ± 9.34	−1.86	0.07
Education (y)	12 (9; 15.75)	15 (10; 16)	−1.06	0.3
**Cause of injury**				
Motor vehicle accident	14 (43.75%)			
Assault	6 (18.75%)			
Fall	7 (21.88%)			
Other	5 (15.62%)			
LOC	9			
Injury and MRI scan interval (hr)	29.25 (16; 57.5)			
TMT-A	47.45 (30.32; 68.92)	43.70 (33.97; 54.50)	−0.62	0.55
TMT-B	136.24 ± 61.24	90.16 ± 36.08	3.11	0.004
DSST	43.96 ± 13.91	56.23 ± 15.65	−3.1	0.003

*mTBI, mild traumatic brain injury; HCs, healthy controls; LOC, loss of concussion; TMT, Trail Making Test; DSST, Digital Symbol Substitution Test*.

**Table 2:** Demographic and clinical characteristics of mTBI patients and HCs.

	**LOC**	**N-LOC**	***t*/*F***	***p***
N	9	9		
Sex	M:4; F:5	M:5; F:4		1
Age	29.44 ± 14.48	28.89 ± 14.10	−0.08	0.94
Education	11.33 ± 2.24	11.22 ± 3.35	−0.08	0.94
TMT-A	56.02 ± 28.26	74.31 ± 58.05	0.81	0.43
TMT-B	129.48 ± 69.15	153.82 ± 55.52	0.73	0.48
DSST	43.14 ± 15.12	41.33 ± 14.05	−0.25	0.81

*mTBI, mild traumatic brain injury; LOC, loss of concussion; TMT, Trail Making Test; DSST, Digital Symbol Substitution Test*.

### MRI Acquisition

All MRI data were acquired on a 3 T MRI scanner (MAGNETOM Skyra, Siemens Healthcare, Erlangen, Germany) in department of radiology, the Second Xiangya Hospital, Central South University, with a 32-channel head coil. The MRI scanning protocols included T1-weighted imaging (T1WI), T2-weighted imaging (T2WI), fluid-attenuated inversion recovery (FLAIR), magnetization-prepared rapid gradient echo (MPRAGE), susceptibility-weighted imaging (SWI), and diffusion MRI. T1WI, T2WI, FLAIR, MPRAGE, and SWI were reviewed for structural abnormalities by two neuroradiologists who had more than 10 years of neuroimaging experience each. Any disagreement between these two observers was resolved by consensus. Diffusion MRI was acquired with TR/TE = 5,400/92 ms, field of view = 224 × 224 mm, 112 × 112 matrix, 40 slices, 2 × 2 × 3 mm voxels, bandwidth = 1,654 Hz/pixel, b = 1,000/2,000 s/mm^2^, 64 diffusion-weighting directions at each *b* value, and 10 b0 scans. All subjects were placed in a supine position with foam padding between their head and the head coil to minimize head motion.

### Neuropsychological Tests

All participants completed the following three cognitive tests: (1) The Digit Symbol Substitution Test (DSST). The DSST has been frequently used to assess participants' processing speed, sustained attention and working memory (33, 34). Patients were shown nine numbers and their corresponding symbols, and then the participants were instructed to match the correct symbol to the corresponding number in one and a half min; (2) The Trail Making Test (TMT) A (35). This test was administered as a baseline measure of motor and visual search speed (36, 37). The subjects were instructed to draw lines connecting consecutively numbered points from 1 to 25 as quickly as possible. The score was the number of seconds required to complete the task, where a shorter time indicated better performance. (3) The TMT-B (35). This test used widely as a measure of set shifting and inhibition (38, 39). The participants were asked to switch alternately between 13 numbers (1–13) and 12 letters (A-L) and connect them in ascending order (1-A-2-B…12-L-13) as quickly as possible. All of these tests have been widely used in neuropsychological assessments as indicators of cognitive processing speed and executive functioning (40). The subjects completed these neuropsychological tests on the same day as the MRI scan.

### Image Analysis

Image processing included initial preprocessing and diffusion metrics computation. Prior to preprocessing, each subject's diffusion images were visually inspected to verify that the images were free from major artifacts (e.g., head motion). motion, eddy current artifacts, and geometric distortion were corrected using the eddy command provided in the FMRIB Software Library (FSL) (41). Using an in-house MATLAB script, the transformation matrices were used. output from the eddy command were used to rotate the corresponding diffusion-weighting directions to match the rotation of the brain image during the motion correction process and then extract the b0 image, and non-brain voxels were masked out by applying the FSL command bet to the subject b0 image. In addition, then DTI metrics were calculated by the FSL command dtifit, fractional anisotropy (FA), axial diffusivity (AD), radial diffusivity (RD) and mean diffusivity (MD) were been detected. DKI model fitting was performed using Diffusion Kurtosis Estimator (DKE: http://academicdepartments.musc.edu/cbi/dki/dke.html) (version 2.6.0). NODDI parameters were calculated using the open-source tool AMICO (github.com/daducci/AMICO) (42).

### Tract-Based Spatial Statistics (TBSS) Analysis

TBSS (43) was performed using the FSL toolbox, TBSS. TBSS extracted a common whole-brain white-matter skeleton in the standard Montreal Neurological Institute (MNI) space to minimize partial volume effects in finite imaging resolution. The WM skeleton included only voxels in the center of WM tracts and excluded edge voxels, which may be contaminated with signals from the nearby anatomical structures. Within the WM skeleton, non-parametric permutation-based statistics were carried out using the FSL *randomize* command for voxelwise statistical analyses and used age as a covariant in this study. Threshold-free cluster enhancement (44) and 5,000 permutations were utilized to obtain a corrected *P*-value. WM voxels were considered significant as a corrected *P*-value <0.05 after being adjusted for multiple comparisons by controlling the familywise error rate.

*Post-hoc* region-of-interest (ROI) analysis: To produce aggregate results at the subject level, *post-hoc* ROI analyses were performed. For each subject, the mean of each diffusion metric was computed in the clusters that tested significant in TBSS. For between-group differences, a boxplot was used with subjects' means plotted according to their group membership. The anatomical interpretation of the ROI was based on the “JHU ICBM-DTI-81 White-Matter Labels” provided in FSL after skeletonization.

### Statistical Analysis

The demographic characteristics and neuropsychological data were analyzed using IBM SPSS Statistics 24.0 for Windows. Unpaired two-sample *t*-tests, chi-square tests, and Kruskal-Wallis tests were performed for age, gender, education, and neuropsychological tests. The correlations between the mean values of diffusion parameters and injury and MRI scan interval as well as neuropsychological tests were evaluated by partial correlation, using age and education as covariates at the whole subject level. Spearman correlation was evaluated at the mTBI patient level. Correlations were corrected for multiple comparisons using a familywise error correction. Receiver operating characteristic (ROC) curve analysis was conducted using MedCalc version 19.5.6 (MedCalc Software bvba, Ostend, Belgium) to compare diffusion metrics to discriminate mTBI patients from controls based on the average value of whole-brain WM. The area under each ROC curve was calculated to determine which area under the curve (AUC) was greatest, and then an ROC curve analysis was conducted to identify a threshold value for a significant diffusion metric to discriminate mTBI patients from HCs. The threshold was identified by maximizing the Youden index.

## Results

### Demographic and Clinical Characteristics

The participant characteristic comparisons are presented in Table 1. The sample included 32 mTBI patients and 31 HCs. There was no statistically significant difference between patients and HCs with regard to sex, age, or education. The median injury and MRI scan interval was 29.25 h. Nine patients had a witnessed period of LOC. The demographic characteristics of the LOC and non-LOC groups are shown in Table 2.

### Diffusion Metrics

**Figure 1:**
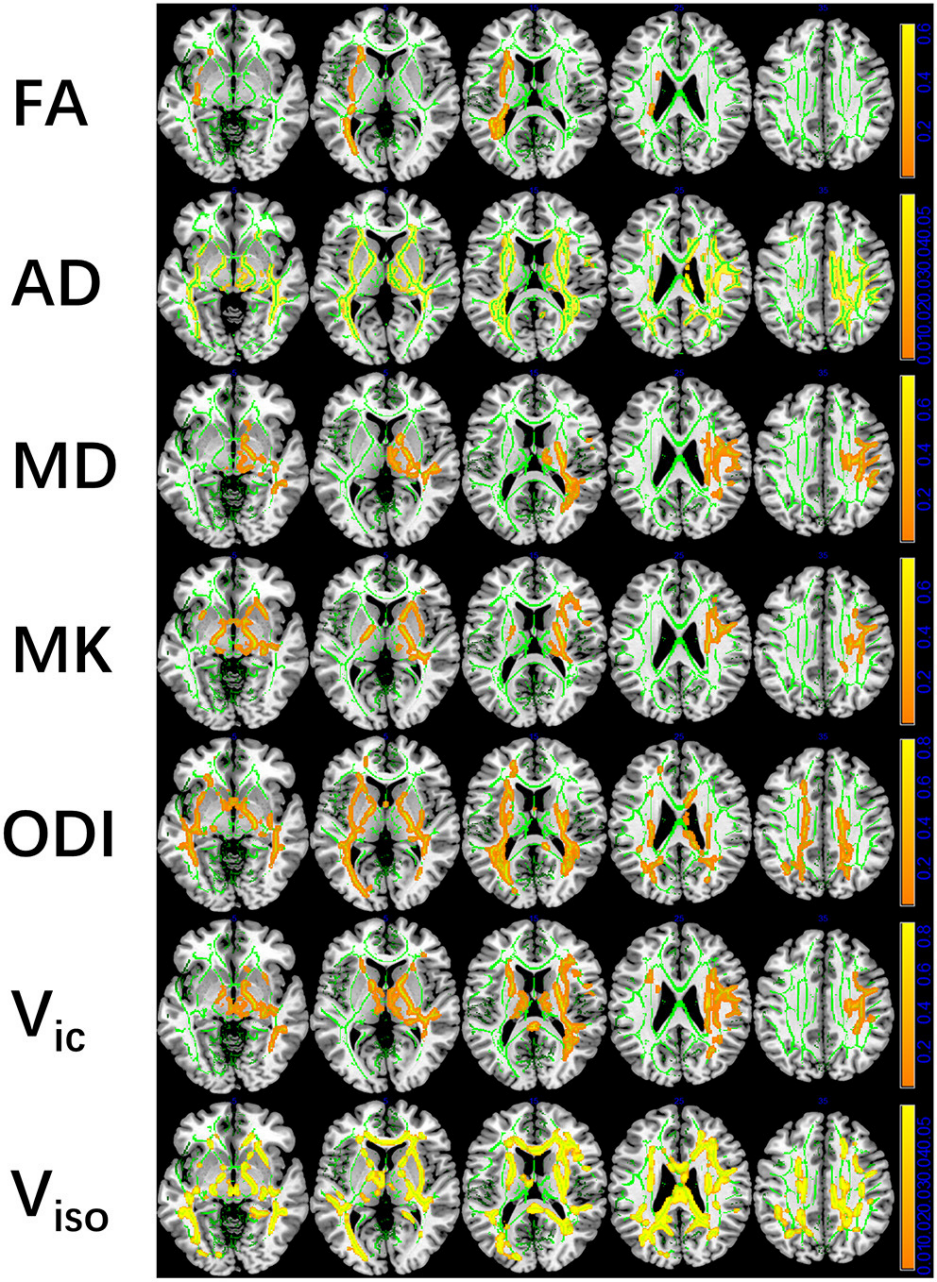
Tract-based spatial statistics (TBSS) results of diffusion metrics. The TBSS analyses revealed increased FA, AD, MD, and decreased MK, ODI, Vic, and Viso in patients compared with controls. Green represents White Matter skeleton. Orange-yellow represents areas of significant differences. Orange represents lower FA, AD, MD, higher MK, ODI, Vic, and Viso, and yellow represents higher FA, AD, MD, lower MK, ODI, Vic, and Viso. FA, fractional anisotropy; AD, axial diffusivity; MD, mean diffusivity; MK, mean kurtosis; ODI, orientation dispersion index; Vic, the volume fraction of intra-cellular water; Viso, the volume fraction of the isotropic diffusion compartment.

**Figure 2:**
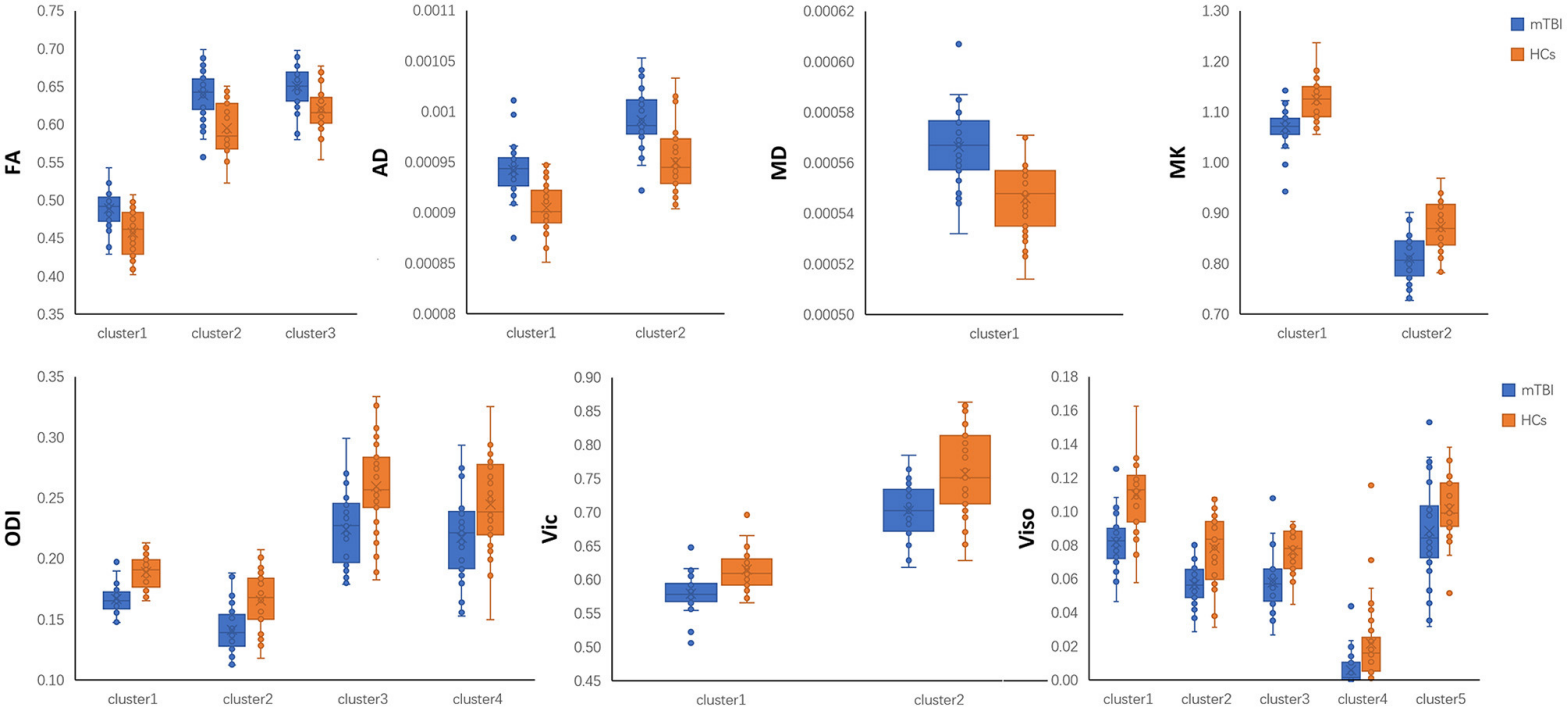
*Post-hoc* region-of-interest (ROI) analysis results. Clusters are significant tracts in Tract-based spatial statistics (TBSS). Different boxplot represent different diffusion metrics. Orange box represent healthy controls (HCs), and blue box represent mild traumatic brain injury (mTBI) group. FA, fractional anisotropy; AD, axial diffusivity; MD, mean diffusivity; MK, mean kurtosis; ODI, orientation dispersion index; Vic, the volume fraction of intra-cellular water; Viso, the volume fraction of the isotropic diffusion compartment.

mTBI vs. HCs: The TBSS analyses revealed higher FA, AD, and MD and lower MK, ODI, Vic and Viso in patients than in controls. FA showed the fewest significant voxels, and Viso showed the most (Table 3). Abnormal diffusion metrics were detected in the following regions: body and splenium of the corpus callosum, internal capsule, external capsule, corona radiata, posterior thalamic radiation and superior longitudinal fasciculus (Figure 1). The abnormality of corpus callosum was only detected by diffusion metrics of NODDI. The results based on ROIs that tested significant in TBSS are shown in Figure 2.

**Table 3:** Anatomical regions of tract-based spatial statistics results.

	**Cluster index**	**Anatomical regions**	**Voxels**	**Min *p***	**X**	**Y**	**Z**
FA	1	External capsule L, Anterior corona radiata L	1,399	0.019	113	149	76
	2	Posterior thalamic radiation (include optic radiation) L, Superior longitudinal fasciculus L	927	0.027	126	70	75
	3	Retrolenticular part of internal capsule L, Posterior corona radiata L	385	0.041	118	95	83
AD	1	Posterior thalamic radiation (include optic radiation) R, External capsule R, Internal capsule R	19,661	0.003	52	82	71
	2	Posterior thalamic radiation (include optic radiation) L, External capsule L, Internal capsule L	7,303	0.007	113	151	81
MD	1	Superior longitudinal fasciculus R, Superior corona radiata R, Posterior limb of internal capsule R	13,755	0.023	62	106	90
MK	1	Internal capsule R, Superior corona radiata R, External capsule R	13,982	0.02	96	94	44
	2	External capsule L	155	0.048	116	137	62
ODI	1	Posterior thalamic radiation (include optic radiation) L & R, Posterior limb of internal capsule L & R, External capsule L & R	16,658	0.001	112	152	69
	2	Superior corona radiata L, Body of corpus callosum	1,249	0.027	108	129	113
	3	Anterior corona radiata L	370	0.031	112	171	84
	4	Corona radiata L (Anterior and Superior part)	187	0.045	109	143	104
Vic	1	Superior longitudinal fasciculus R, internal capsule R	17,254	0.012	65	112	82
	2	Splenium of corpus callosum	112	0.048	91	87	82
Viso	1	Corona radiata R, External capsule R, corpus callosum	17,699	0.003	63	104	81
	2	Posterior thalamic radiation (include optic radiation) L, Superior longitudinal fasciculus L, Splenium of corpus callosum	7,011	0.027	131	82	83
	3	Anterior limb of internal capsule L, corona radiata L(Anterior and Superior part)	2,178	0.038	117	146	97
	4	External capsule L	68	0.049	120	132	62
	5	Superior corona radiata L	60	0.049	115	114	103

*FA, fractional anisotropy; AD, axial diffusivity; MD, mean diffusivity; MK, mean kurtosis; ODI, orientation dispersion index; Vic, the volume fraction of intra-cellular water; Viso, the volume fraction of the isotropic diffusion compartment*.

### Diffusion Metrics

LOC vs. non-LOC: The TBSS analyses did not reveal any statistically significant differences in DTI, DKI or NODDI metrics between LOC and non-LOC patients.

### Neuropsychological Tests Results

TMT-A data were unavailable in 5 mTBI patients because three patients had hand fractures and two patients needed to stay in a reclining position. TMT-B data were unavailable in 10 mTBI patients and seven healthy controls for the same reasons as TMT-A, and five patients and seven controls forgot the order of the alphabet, resulting in an unusually long test time. DSST data were lost in 6 mTBI patients; five of them had the same reasons as before, and the other patient had arm fractures. Compared with healthy controls, the performance of TMT-B and DSST tests was significantly worse in patients with mTBI (Table 1). There was no significant difference between LOC and non-LOC patients.

### Correlation Results

**Figure 3:**
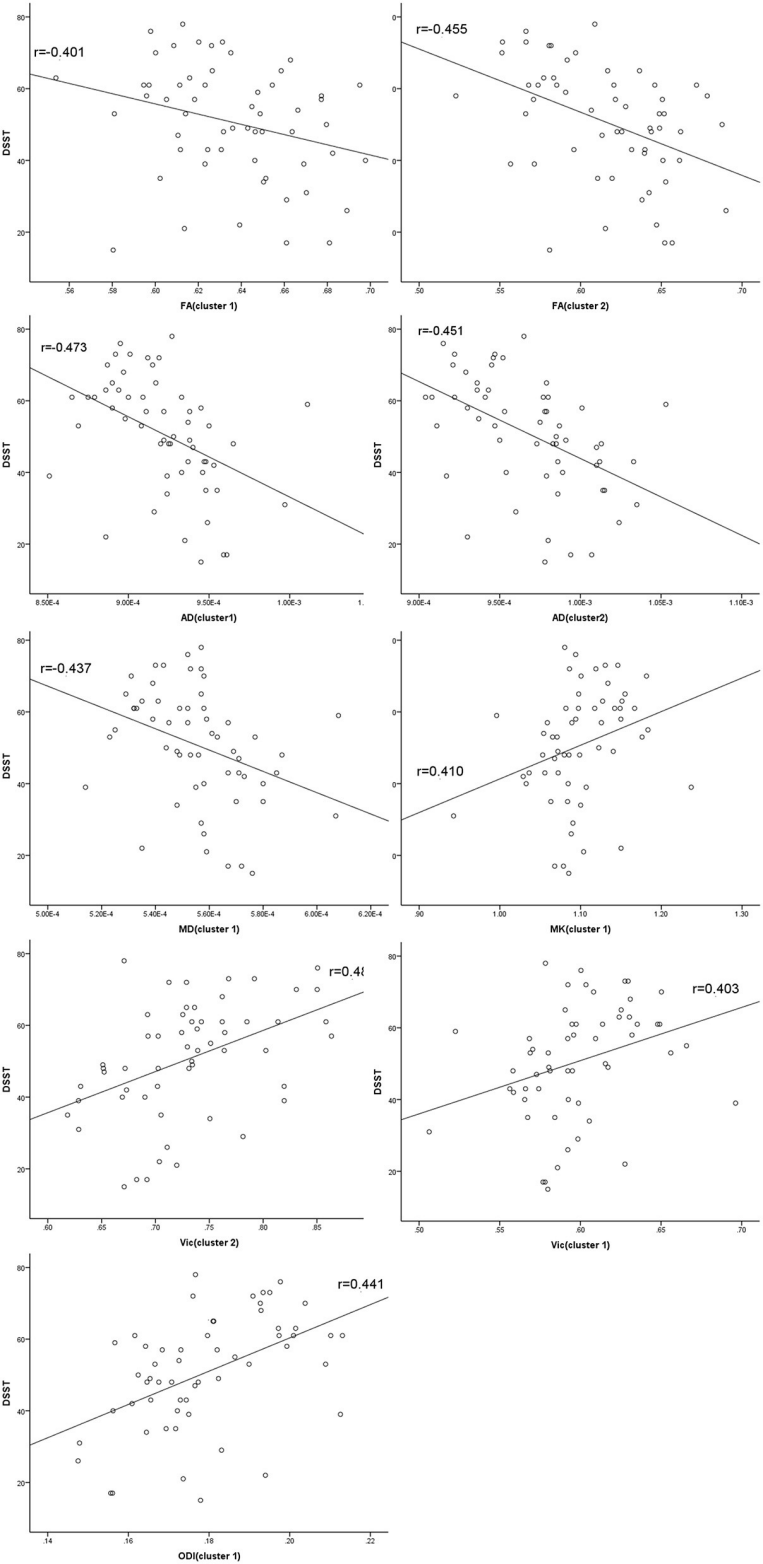
Partial correlations between diffusion metrics and cognitive function. Clusters are significant tracts in Tract-based spatial statistics (TBSS). FA in cluster 1 and 2, AD in cluster 1 and 2 and MD in cluster 1 correlated negatively with the Digital Symbol Substitution Test (DSST) scores, and MK in cluster 1, ODI in cluster 1 and Vic in cluster 1 and 2 correlated positively with DSST scores. FA, fractional anisotropy; AD, axial diffusivity; MD, mean diffusivity; MK, mean kurtosis; ODI, orientation dispersion index; Vic, the volume fraction of intra-cellular water.

**Figure 4:**
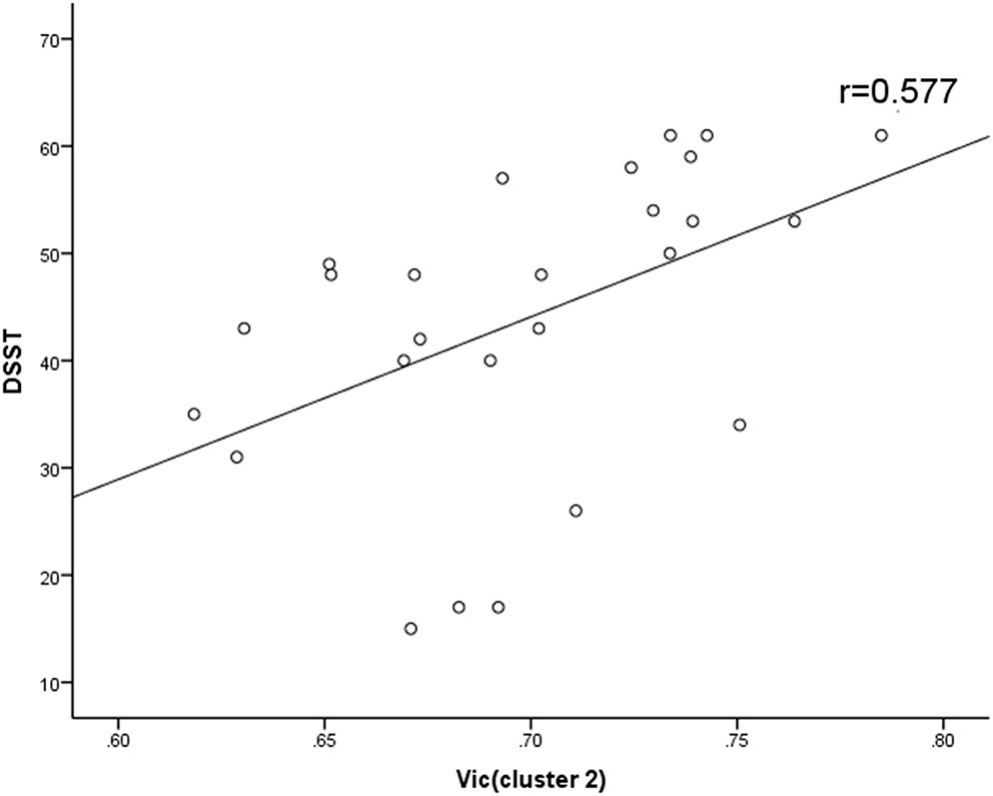
Spearman correlations between Vic and DSST in mTBI group. Vic in cluster 2 correlated positively with DSST scores. Vic, the volume fraction of intra-cellular water.

At the level of the overall sample, FA in clusters 1 and 2, AD in cluster 1 and 2 and MD in cluster 1 were negatively correlated with DSST scores, and MK in cluster 1, ODI in cluster 1 and Vic in clusters 1 and 2 were positively correlated with DSST scores (Table 4). The correlation between Vic of the splenium of the corpus callosum and DSST scores showed the smallest *p*-value (*p* = 0.000, *r* = 0.481). At the mTBI group level, Vic in cluster 2 was positively correlated with DSST scores (*p* = 0.002, *r* = 0.577). Furthermore, diffusion metrics did not correlate with injury or MRI scan interval in either the total sample or the mTBI group. The partial correlation between diffusion metrics and DSST scores is presented in Figure 3. The correlation between Vic and DSST scores in the mTBI group is presented in Figure 4.

**Table 4:** Partial correlation results between diffusion metrics and cognitive function.

	**FA (cluster 1)**	**FA (cluster 2)**	**AD (cluster 1)**	**AD (cluster 2)**	**MD (cluster 1)**	**MK (cluster 1)**	**ODI (cluster 1)**	**Vic (cluster 1)**	**Vic (cluster 2)**
DSST	*r* = −0.401	*r* = −0.455	*r* = −0.473	*r* = −0.451	*r* = −0.437	*r* = 0.410	*r* = 0.441	*r* = 0.403	*r* = 0.481
	*p* = 0.001	*p* = 0.000	*p = 0.000*	*p = 0.001*	*p* = 0.001	*p* = 0.002	*p* = 0.001	*p* = 0.002	*p* =0.000

*FA, fractional anisotropy; AD, axial diffusivity; MD, mean diffusivity; MK, mean kurtosis; ODI, orientation dispersion index; Vic, the volume fraction of intra-cellular water; DSST, Digital Symbol Substitution Test*.

### ROC Results

**Figure 5:**
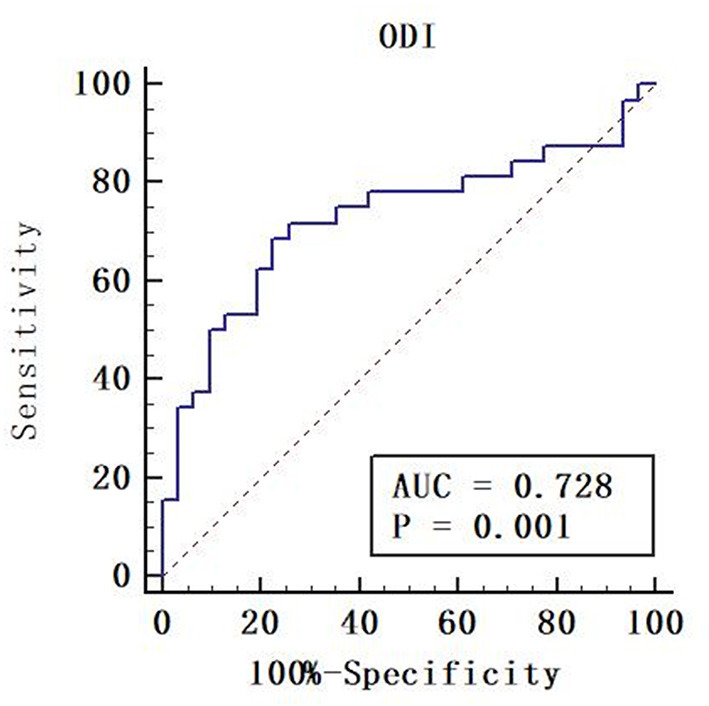
Receiver operating characteristic (ROC) curve of orientation dispersion index (ODI). Areas under the ROC curve (AUC) is 0.728. The cut-off value was ODI = 0.168 with a sensitivity of 50.0%, a specificity of 90.3%.

ODI had the largest area (AUC = 0.728) (Table 5). Although there was no significant difference, the diffusion metrics of NODDI showed larger AUCs than those of DTI and DKI, except for the AUC comparison between FA and ODI (*p* = 0.0264, Z = 2.221). For ODI, the optimal cutoff value was 0.168, with a sensitivity of 50.0 and a specificity of 90.3%. The ROC curve of ODI is shown in Figure 5.

**Table 5:** Areas under the ROC curve (AUC) of diffusion metrics.

	**AUC**	**SE**	**95% CI**
FA	0.567	0.0747	0.436–0.691
AD	0.723	0.0657	0.596–0.829
MD	0.636	0.0711	0.505–0.753
MK	0.655	0.0697	0.525–0.771
ODI	0.728	0.0669	0.601–0.832
VIC	0.67	0.0695	0.540–0.784
VISO	0.71	0.0674	0.582–0.817

*ROC, receiver operating characteristic; SE, standard error; CI, confidence interval; FA, fractional anisotropy; AD, axial diffusivity; MD, mean diffusivity; MK, mean kurtosis; ODI, orientation dispersion index; Vic, the volume fraction of intra-cellular water; Viso, the volume fraction of the isotropic diffusion compartment*.

## Discussion

mTBI patients showed WM abnormalities and cognitive dificit compared to HCs in the acute stage. DSST is a superior reflection of brain microstructural changes after mTBI, especially its correlation with Vic in the splenium of the corpus callosum.

Long tract fibers (corona radiata, posterior thalamic radiation, and superior longitudinal fasciculus) and commissural fibers (corpus callosum) are most vulnerable to damage (45, 46) because of their long length and high membrane-to-cytoplasmic ratios (47). Our study obtained similar results and corroborated previous findings that the posterior region of the corpus callosum was more vulnerable to injury than the anterior region (48, 49). Only the diffusion metrics of NODDI characterize the abnormality of the corpus callosum, indicating the important role of NODDI to investigate key injury region after mTBI.

Changes in diffusion parameters indicated WM abnormalities in our research. First, increased FA was reported frequently within 2 weeks after injury, (50) which was believed to reflect injury-related cytotoxic edema (51) or reactive astrogliosis (52). Second, increased AD were thought to indicate brain edema. Third, MD increased when WM was disorganized or damaged (53). The result of increased FA, MD, and rather an unchanged RD was supported by Veeramuthu et al. (46) indicate that the supporting cells like astrocytes migrate to the site of injury, and caused the increase of cells density and diffusivity. Fourth, decreased MK was reported in a few studies on mTBI patients, and it was probably associated with degenerative changes and neuronal shrinkage (12, 14, 20, 54–57). Furthermore, decreased ODI indicated that WM fibers tended to be parallel, which is consistent with the FA results in our study. In a longitudinal study (24), ODI increased over time; we are interested in investigating the long-term effect in our future research. Finally, Vic, as a potential proxy for axonal density measurements, may be explained by edema and axonal beading followed by apoptosis (58) in our study. The primary effect on WM after mTBI is increased extra neurite water volume, which may be caused by the mixed effects of intra-neurite and isotropic water volume. This may be the reason for the discrepancy between our study and others (23–25). Combining the changes of three diffusion metrics of FA, ODI, and Vic, we infer that in the acute stage of mTBI, the WM fibers of the subjects tend to be parallel and loose. And the change of FA value reflects that the effect of paralleled WM fiber is greater than the influence of WM porosity. Since the corpus callosum is parallel fibers, and the influence of WM fiber looseness is not enough to cause a significant result, only the metrics of NODDI can detect the change of corpus callosum. As shown in our results, the combination of FA and MD showed changes in the whole brain. The NODDI parameters showed more spatially extensive effects than the DTI or DKI parameters, which was consistent with the ROC results. The aforementioned results indicated that NODDI was more sensitive to the effects of mTBI. Moreover, according to the results of our research and previous studies, MK and Vic were the most stable parameters of mTBI patients in terms of short-term effects, showing the functional complementarity of DKI and NODDI to DTI.

Processing speed is the fundamental cognitive process to support higher cognitive functions (59), such as sustained attention and working memory detected by the DSST. A previous study demonstrated that the external capsule conveys fibers from the cognitive region of the cerebral cortex to the striatum and contributes to cognitive control (60). Furthermore, the superior longitudinal fasciculus and corona radiata are structures critical to working memory (61). The internal capsule is related to disorders of attention (62, 63). Moreover, the corpus callosum, as the commissural fiber that connects the two hemispheres, participates in the process of attention and memory (60). These prior studies corroborate our results showing that WM abnormalities correlate to cognitive decline.

The negative correlation between FA and the DSST showed that the higher the FA was, the worse the cognitive function. Previous studies have found a similar result (50, 61, 64). This suggests that understanding the relationship between DSST and FA is important for patient management, such as addressing cytotoxic edema and reactive astrogliosis. The correlations between AD, MD, MK, and Vic, and DSST scores are supported by others (19, 23, 65). Lower Vic indicates higher axonal density and is accompanied by worse cognitive performance. A lower axonal density indicated a larger interstitial space and a larger buffering effect. At the same time, there are more neuro-supportive cells in the interstitial space (23) that help preserve brain function after TBI (66). There was no correlation between cognitive function and Viso, indicating that the change in free water does not affect cognitive function.

This study has some limitations. First, the moderate sample size, particularly the small sample sizes of the subgroups, may have been the cause of the negative results between the LOC and non-LOC groups. More mTBI patients should be recruited in future studies to test and clarify the results of the present research. Second, DSST and TMT cannot specifically detect a particular aspect of cognitive function. There are many brain regions associated with DSST. We could not provide an exact correlation between brain regions and the particular aspect of cognitive function. Many other tests should be performed to detect these correlations. Third, concomitant injury to other body regions confounded the performance of mTBI patients, although we minimized the inclusion of such patients. One of the future directions of our work will be the inclusion of a trauma control group. Finally, to determine the true predictive utility of these diffusion metrics, longitudinal studies should be performed to assess changes in imaging measures over time.

## Conclusion

The diffusion parameters of DTI, DKI, and NODDI showed abnormalities in WM and their relationship with changes in cognitive function. The superior voxel count of NODDI and the consistency of MK, and Vic in previous studies and our study showed the functional complementarity of DKI and NODDI to DTI.

## Data Availability Statement

The raw data supporting the conclusions of this article will be made available by the authors, without undue reservation.

## Ethics Statement

The studies involving human participants were reviewed and approved by the Ethics Committee of the Second Xiangya Hospital, Central South University. The patients/participants provided their written informed consent to participate in this study.

## Author Contributions

JL: conceptualization, supervision, project administration, and funding acquisition. SH, ML, HZ, and JL: methodology. SH and HZ: software and visualization. SH, CH, ML, and HZ: validation. SH and CH: formal analysis and investigation. SH, CH, and ML: resources and data curation. SH: writing –original draft preparation. SH, CH, ML, HZ, and JL: writing –review and editing. All authors have read and agreed to the published version of the manuscript.

## Funding

This research was funded by the National Natural Science Foundation of China (81671671), the Science and Technology Project of Changsha (kq1801115), the Central South University (2021gfcx05), and Clinical Research Center for Medical Imaging in Hunan Province (2020SK4001).

## Conflict of Interest

HZ is employed by MR Scientific Marketing, Siemens Healthcare Ltd. The remaining authors declare that the research was conducted in the absence of any commercial or financial relationships that could be construed as a potential conflict of interest.

## Publisher's Note

All claims expressed in this article are solely those of the authors and do not necessarily represent those of their affiliated organizations, or those of the publisher, the editors and the reviewers. Any product that may be evaluated in this article, or claim that may be made by its manufacturer, is not guaranteed or endorsed by the publisher.
